# Pediatric Solid-State 3D Models of Lumbar Vertebrae and Spine

**DOI:** 10.7759/cureus.58938

**Published:** 2024-04-24

**Authors:** Olena Bolgova, Volodymyr Mavrych

**Affiliations:** 1 Anatomy and Genetics, College of Medicine Alfaisal University, Riyadh, SAU; 2 Anatomical Sciences, College of Medicine Alfaisal University, Riyadh, SAU

**Keywords:** virtual reality, medical education, lumbar spine, solid-state 3d models, pediatric lumbar vertebrae

## Abstract

Introduction

While various 3D vertebral models have been utilized in numerous studies, there is a notable gap in the representation of pediatric lumbar vertebrae and spine. This study aimed to describe the changing shapes of lumbar vertebrae and spine with age and to develop precise 3D models.

Materials and methods

Solid-state 3D models of pediatric lumbar vertebrae and spine were created using SOLIDWORKS® Simulation software for five age groups: newborns, infants (ages 0-1), toddlers (ages 1-3), middle childhood (ages 4-7), and preadolescents (ages 8-12). Models were composed of components with varying biomechanical characteristics.

Results

Created 3D models replicate variations in the dimensions and configurations of vertebrae, taking into account osteometric analyses conducted on actual vertebral specimens. These models also include elements made of cartilage, representing various phases of vertebral growth during ontogeny. Additionally, through 3D parametric design, we developed comprehensive lumbar spine models, incorporating both the vertebrae and intervertebral disks.

Conclusion

Created pediatric solid-state vertebral 3D models can be utilized in developing virtual or augmented reality applications and for medical research. Users can interact with models, allowing virtual exploration and manipulation, enhancing learning experiences and facilitating a better understanding of spatial relationships. These solid-state 3D models allow finite element analysis and can be used for further research to calculate internal relative deformations and stress distribution under different conditions.

## Introduction

Accurate three-dimensional (3D) lumbar spine reconstruction from various imaging modalities such as CT scans, MRI, X-rays, and ultrasound plays a crucial role in clinical practice and research. These reconstructions provide detailed anatomical information essential for diagnosis, treatment planning, and biomechanical analysis of spinal pathologies and conditions.

CT scans provide high-resolution 3D models that enable precise visualization of bone structures, which are beneficial for assessing fractures, tumors, or degenerative changes in the lumbar spine. MRI offers detailed soft tissue visualization, diagnosing spinal cord compression, disc herniation, and other soft tissue abnormalities. A deep learning algorithm has been discussed to generate 3D lumbar spine CT images exclusively from MRI data, potentially reducing patient radiation exposure and healthcare burdens. The study compares the accuracy of synthetic CT images produced by the model, showing promising results in clinically relevant measurements [1].

MRI is known for its costliness and time consumption, while CT scans may expose patients to high radiation doses. X-rays are a quick and cost-effective method for evaluating spinal alignment, fractures, and degenerative changes in the lumbar spine despite the lack of 3D visualization. A technique has been presented to reconstruct scaled 3D lumbar vertebral models from a single 2D fluoroscopic image and statistical shape modeling, demonstrating an average error of 1.0 mm for lumbar vertebrae [2].

In recent years, musculoskeletal ultrasound has emerged as a non-invasive and real-time imaging modality for evaluating bone and soft tissues in the lumbar spine. Its portability, cost-effectiveness, and ability to provide dynamic imaging make it a valuable tool for assessing spinal conditions, especially in clinical settings where traditional imaging modalities may be limited. A recent study explores the use of musculoskeletal ultrasound for non-invasive 3D bone modeling of lumbar vertebrae as an accessible and cost-effective alternative to traditional imaging methods, compares the reliability of distances measured on CT-scan and ultrasound-derived 3D models, highlights the potential of ultrasound for high-resolution, real-time imaging of bone structures [3]. Overall, 3D lumbar spine reconstruction from CT scans, MRI, X-rays, and ultrasound facilitates comprehensive and multidimensional spinal anatomy and pathology evaluation. These imaging techniques are crucial in guiding treatment decisions, monitoring progression, and improving patient outcomes in individuals with lumbar spine disorders.

3D reconstruction of the human spine from various imaging modalities is currently utilized for examining spinal morphology and understanding demographic, sex, and age-related variations in vertebral structures. It was found that the shape of the human lumbar spine is influenced by various factors such as erect posture, sexual dimorphism, and genetic background. In a study, 3D geometric morphometrics were utilized to analyze lumbar spine morphology in different populations, revealing differences in lumbar lordosis between Mediterranean and South African samples and distinct variations in lumbar spine features between males and females [4]. Another study demonstrated the use of CT scan-derived 3D models of lumbar vertebrae for sex identification, achieving high accuracies [5]. Furthermore, it evaluated age-related changes in the vertebral body using 3D postmortem CT images, proposing an age estimation formula based on volumes and ratios derived from the fourth lumbar vertebral body [6].

Morphological spine studies demonstrate the significance of advancing 3D imaging techniques for enhancing our understanding of spinal anatomy and biomechanics and optimizing device design and spinal interventions. A study explored the 3D shape of lumbar vertebral end plates in normal adult spines and their correlation with factors such as age, gender, and end plate characteristics [7]. The research revealed distinct differences between superior and inferior end plates, with superior end plate shapes varying significantly across different spinal levels. Insights from this study suggest potential implications for future interbody device designs. In a separate study, a method was developed to accurately represent spinal sagittal alignment using 3D models constructed from CT scans and standing radiographs, offering a reproducible approach for creating patient-specific spinal geometries [8].

Additionally, a parametric modeling method for 3D representing intervertebral disc spaces based on CT imaging enables detailed morphometric analysis and precise characterization of disc morphology [9]. Furthermore, an investigation into lumbar facet joint motion characteristics and the impact of weight-bearing in the sitting position provides insights into asymmetry and movement patterns of facet joints in different lumbar segments [10].

A comprehensive understanding of vertebral fractures and lumbar spine segmentation is essential for effective diagnosis and treatment planning. Using statistical classification analysis, quantitative 3D vertebral morphometry by parametric modeling of vertebral bodies was conducted to accurately differentiate between normal and fractured vertebral bodies [11]. The study highlighted clinically meaningful morphometric features that distinguish between normal and fractured vertebral bodies, offering a valuable tool for diagnosing and predicting vertebral fractures, particularly in individuals at risk of osteoporosis. A related study proposed a deep learning-based solution for the automatic location and segmentation of lumbar vertebral body cancellous bone [12]. The approach combines the strengths of convolutional neural networks and vision transformers to address anatomical variations and scarcity of data, resulting in improved segmentation accuracy and performance compared to existing methods. Additionally, maps of traumatic thoracolumbar fractures through 3D reconstruction were developed, providing insights into their patterns and characteristics and enhancing clinical decision-making [13].

Furthermore, an artificial intelligence-based MRI segmentation method for constructing a 3D model of lumbosacral structures was introduced, aiding in selecting percutaneous endoscopic lumbar discectomy approaches at the L5/S1 level [14]. The study demonstrated high accuracy in segmenting key anatomical structures, facilitating preoperative assessment approaches.

The planning of vertebral surgery encompasses various aspects of research, innovation, and patient education to improve surgical outcomes and enhance patient understanding. A study was conducted collecting morphometric data on lumbar vertebrae pedicles in the eastern Indian population, essential for optimizing the free-hand technique of pedicle screw insertion [15]. Focus was placed on creating personalized 3D digital models for selective dorsal rhizotomy surgeries, utilizing innovative techniques to enhance preoperative planning and surgical navigation [16]. The SpineBox, a cost-effective and open-access simulator for spinal anatomy and pedicle screw placement, was introduced, catering to neurosurgical trainees globally [17].

The value of personalized 3D printed models in patient education for degenerative lumbar diseases was highlighted, demonstrating improved understanding and satisfaction compared to traditional imaging methods [18]. Overall, these studies exemplify the integration of advanced technologies and patient-centric approaches in the planning and executing vertebral surgeries, paving the way for enhanced surgical outcomes and patient care.

While various 3D models have been utilized in different research studies, there remains a notable gap in the representation of pediatric lumbar vertebral structures. This study aimed to describe how pediatric lumbar vertebrae and spine shapes vary with age and to develop precise solid-state 3D models of these structures.

## Materials and methods

General features of the models

Lumbar vertebra solid-state 3D models were developed through three-dimensional parametric design in SOLIDWORKS® Simulation software (https://www.solidworks.com/) by emulating lumbar vertebrae based on specimens from the Museum at the Department of Human Anatomy, Luhansk State Medical University. A detailed osteometric analysis of the lumbar spine was conducted in our previous research for different age groups [19]. This study utilized the mean measurements of 57 lumbar vertebrae from the first five age groups (Table 1). The primary goal of this research was crafting vertebral 3D models that maintain functional integrity, mirrored the principal anatomical features, and were composed of geometric shapes to simplify finite element analysis.

**Table 1:** Mean dimensions of the lumbar vertebrae (MM) AVBH – anterior vertebral body height, PVBH – posterior vertebral body height, SVBL – superior vertebral body length, IVBL – inferior vertebral body length, SVBW – superior vertebral body width, MVBW – middle vertebral body width, IVBW – inferior vertebral body width

Age group	Dimension	L1	L2	L3	L4	L5
(1) 0 yrs.	AVBH	6.9	7.0	7.2	7.1	7.0
n = 23	PVBH	7.2	7.2	7.2	7.3	7.3
	SVBL	7.6	7.6	7.7	7.8	7.8
	IVBL	7.7	7.7	7.7	7.8	7.8
	SVBW	14.5	14.6	14.6	14.8	14.8
	MVBW	15.1	15.1	15.2	15.4	15.4
	IVBW	14.7	14.6	14.6	14.9	14.9
(2) 0-1 yrs.	AVBH	8.4	8.4	8.5	8.5	8.4
n = 12	PVBH	8.6	8.6	8.7	8.7	8.7
	SVBL	9.4	9.5	9.5	9.6	9.6
	IVBL	9.4	9.6	9.5	9.6	9.6
	SVBW	17.3	17.3	17.4	17.5	17.6
	MVBW	17.6	17.8	17.8	17.9	18.0
	IVBW	17.2	17.6	17.4	17.6	17.7
(3) 1-3 yrs.	AVBH	11.7	11.7	12.1	12.5	12.5
n = 9	PVBH	11.2	11.1	11.1	11.3	10.8
	SVBL	17.4	17.2	17.5	17.7	17.2
	IVBL	16.3	16.3	16.2	16.2	16.3
	SVBW	28.9	28.7	29.2	29.5	28.6
	MVBW	27.6	27.4	27.6	27.7	27.4
	IVBW	27.2	27.2	27.0	26.9	27.1
(4) 3-7 yrs.	AVBH	16.1	17.6	18.5	17.0	16.7
n = 7	PVBH	16.3	15.9	16.1	14.7	13.9
	SVBL	20.9	22.0	22.9	23.2	24.9
	IVBL	22.6	23.2	24.5	24.7	23.1
	SVBW	31.4	33.0	34.4	34.8	36.5
	MVBW	32.2	33.4	35.1	35.4	34.8
	IVBW	33.9	34.9	36.8	37.1	34.0
(5) 8-12 yrs.	AVBH	18.9	20.3	23.0	19.8	20.3
n = 6	PVBH	18.3	19.9	18.2	16.5	16.3
	SVBL	24.6	26.0	28.8	28.8	28.3
	IVBL	25.6	28.5	27.4	28.0	26.5
	SVBW	32.8	34.7	38.4	38.4	37.8
	MVBW	32.5	35.5	36.6	37.0	35.7
	IVBW	34.1	38.0	36.5	37.3	35.3

The hallmark of these models is their straightforwardness and practicality. These foundational principles were employed to assemble models across various age groups. While they are unsuitable for operative planning on individual patients, they enable theoretical exploration of various factors impacting the spine, aid in devising new surgical techniques, and offer predictions on the performance of different endoprostheses based on shape and material.

3D model of a lumbar vertebra

**Figure 1:**
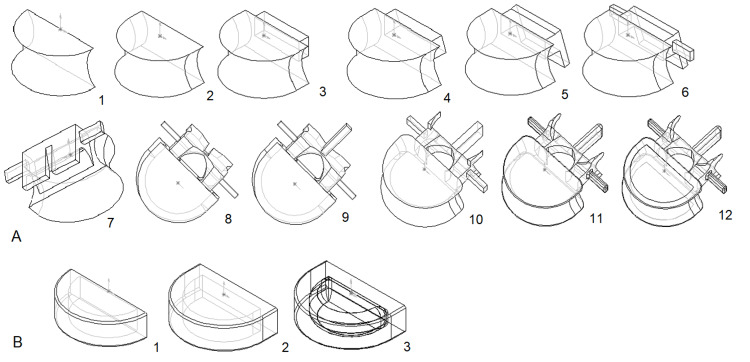
A - The main steps of creating a 3D model of a lumbar vertebra, B - 3D model of an intervertebral disk (explanations are in the text).

The methodology we used for creating the 3D computer model of a lumbar vertebra employed the following steps (Figure 1A):

1. The process was initiated by sketching a closed contour on a plane utilizing the vertebral body's upper, middle, and lower widths and posterior height and then crafting the vertebral body's anterior part through a rotation technique.

2. Subsequently, we lengthened the vertebral body anteroposterior using the upper and lower sagittal diameters.

3. We then constructed a parallelepiped on the vertebral body's posterior aspect from the pedicles' height and width measurements gleaned from the vertebral arch.

4. Using the proportions of the anterior and posterior heights, we calculated and fashioned the inclines on the vertebral body's upper and lower planes and the arch pedicles' posterior edges.

5. Following this, we augmented the vertebral arch and the inferior articular processes alongside the posterior pedicle edge, informed by the lengths previously noted.

6. We then engineered the transverse processes as parallelepipeds situated on the arch's lateral aspect, applying measurements relating to their lengths.

7. Next, the foundation for the spinous process, a notch on the inferior surface of the vertebral arch, was modeled.

8. After this, the vertebral foramen was sculpted, factoring in frontal and sagittal diameter data from osteometric assessment.

9. The spinous process was modeled as a subtly downward-slanting parallelepiped, informed by length metrics.

10. The superior articular processes were crafted next, with consideration for their lengths; we also calculated the internal cutout's diameter to ensure the joint surfaces had a gap no wider than 0.5 mm.

11. Subsequent efforts involved smoothing the sharp edges and angles to give the model its near-final appearance.

12. Finally, the endplates' bony limbus and central fossae were created through cutouts with softened edges on the vertebral body's upper and lower surfaces.

3D model of an intervertebral disk

For constructing three-dimensional models of intervertebral discs, the procedure was notably more straightforward, consisting of just three essential steps (Figure 1B):

1. The anterior segment of the disc was formed using the rotational approach, taking into account the disc's wedge shape and an average inclination angle of 7º. It includes an inner cavity for the nucleus pulposus.

2. Subsequently, the disc's posterior part and an internal recess were developed - this continuation of the cavity houses roughly 50% of the intervertebral disc's volume.

3. In the final phase, we modeled the nucleus pulposus, which was created separately and inserted into the annulus fibrosus cavity.

Material properties

Table 2 shows the biomechanical properties of the different model components we used, taken from the literature data and previous biomechanical studies [20-23].

**Table 2:** Biomechanical properties for different components of 3D models

3D model component	Young's modulus (E), MPa	Poisson's ratio (ν)
Cortical bone	12 000	0.30
Trabecular bone	100	0.20
Cartilage	10	0.40
Nucleus pulposus	1	0.49
Annulus fibrosus	4.2	0.45

The lumbar spine has a segmental arrangement and consists of a series of similar types and shape units - vertebrae and intervertebral disks. To study the basic movements, it is sufficient to simulate actions in a 3D model of one vertebral segment. On the other hand, it is essential to check the propagation of mechanical loads from one segment to another segment to determine the details of the lumbar spine's stress and deformation states when performing various movements under static compression. Therefore, we created 3D computer models of the lumbar spine for children of different age groups.

## Results

The structure of human vertebrae has undergone considerable transformation during human evolution and individual growth. These changes have been both phylogenetic, relating to the species' evolutionary development, and ontogenetic, concerning the development of the individual from embryo to adulthood.

Phylogenetically, the changes in vertebrae reflect adaptions to bipedalism, which have ramifications for balance, locomotion, and the ability to bear weight upright. The lumbar region, for example, has evolved to provide more support and flexibility, allowing for the range of movement required for a bipedal gait. At the same time, the cervical vertebrae have adapted to support the head in a more vertical alignment.

**Figure 2:**
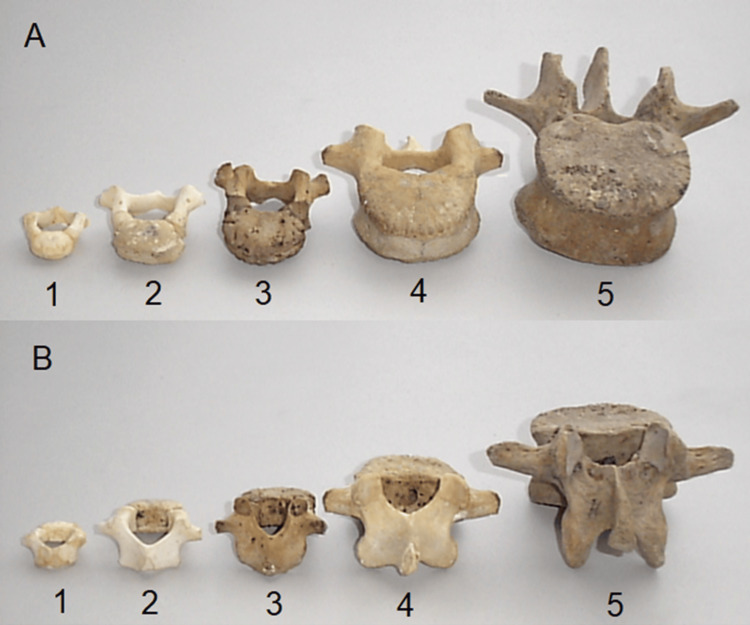
Specimens of the third lumbar vertebra from five age groups (1-5): A - anterior view, B - posterior view.

Ontogenetically, the vertebrae also exhibit significant transformations from the softer, more pliable cartilaginous forms seen in infants to the harder, fully ossified bones that make up the adult spine. During this development, the vertebral growth plates play a critical role in increasing length and size, leading to the final shape and structure of the mature vertebrae (Figure 2).

3D vertebral models of group 1 - Newborns

**Figure 3:**
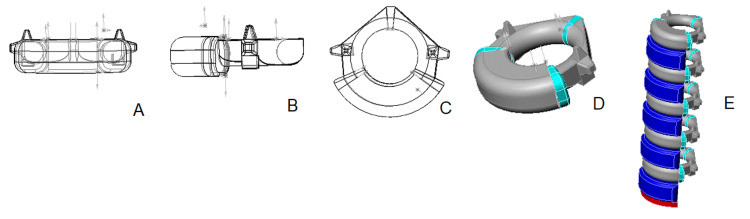
3D model of the lumbar vertebra and spine of group 1: A - anterior view; B - lateral view; C - superior view; D - isometric projection; E - assembled lumbar spine. Color scheme: gray - bone, light blue - cartilage, navy blue - intervertebral discs, red - upper endplate S1.

Three-dimensional models of newborn lumbar vertebrae comprise six elements: three osseous components - the central vertebral body and two semi-arches. Cartilaginous layers connect these bony structures, specifically through the pedicles of the vertebral arch and the juncture where both semi-arches meet along the midline (Figure 3). The vertebral body is characterized by its oval configuration. All extending structures are modeled as truncated pyramidal shapes, with the vertebral foramen presenting as circular. Morphologically, the vertebrae within the newborn lumbar spine model share a uniform architecture.

At this stage, the curvature of lumbar lordosis is not yet defined. The intervertebral discs are created with heights that match those of the vertebral bodies. As shown in Figure 3E, the newborn lumbar spine model is built using average dimensions corresponding to vertebrae and intervertebral discs typical of this age group. It comprises five lumbar vertebrae (L1 through L5), the top endplate of the sacrum (S1), and five intervertebral discs situated between these vertebrae (L1/2 through L5/S1).

3D vertebral models of group 2 - Infants (ages 0-1)

**Figure 4:**
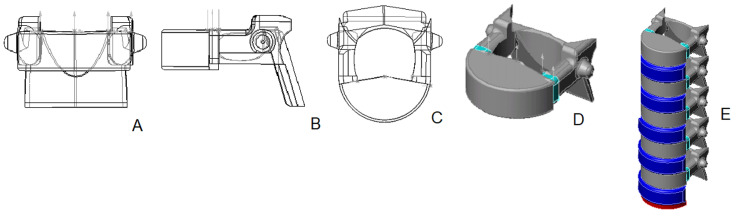
3D model of the lumbar vertebra and spine of group 2: A - anterior view; B - lateral view; C - superior view; D - isometric projection; E - assembled lumbar spine. Color scheme: gray - bone, light blue - cartilage, navy blue - intervertebral discs, red - upper endplate S1.

The 3D models of lumbar vertebrae for infants aged 10 days to 12 months are characterized by four primary components: two bony elements - the central vertebral body and a single vertebral arch (the cartilaginous layer that separates the semiarches undergoes ossification in the first year, except for at L5), and two cartilaginous elements - the vertebral pedicles, which bridge the gap between the body and the arch (Figure 4). The vertebral body is fashioned similarly to a sector of a disc, its peripheries gently rounded. The vertebral arch and foramen are substantially larger than the previous model. The arch resembles an elongated plate, bent at an angle, and houses the facets for the lower articular processes, while the facets for the upper articular processes rest atop it. The spinous process is not yet prominent, appearing as a small angle where the two semi-arches conjoin, and the transverse processes are still rudimentary. Compared to its earlier stages, the cartilaginous pedicles are narrower.

Despite minor differences, all lumbar vertebrae maintain a consistent shape, with the intervertebral disc heights shorter than those of the vertebral bodies. The lumbar lordosis remains undefined. The 3D model of an infant's lumbar spine is comprised of five vertebrae, five intervertebral discs, and the superior endplate of S1, shown in Figure 4E.

3D vertebral models of group 3 - Toddlers (ages 1-3)

**Figure 5:**
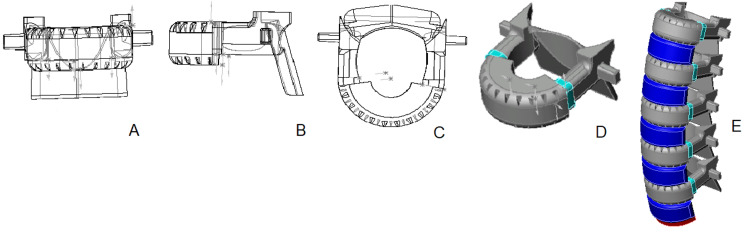
3D model of the lumbar vertebra and spine of group 3: A - anterior view; B - lateral view; C - superior view; D - isometric projection; E - assembled lumbar spine. Color scheme: gray - bone, light blue - cartilage, navy blue - intervertebral discs, red - upper endplate S1.

For children 1-3 years, we constructed 3D models consisting of four components. This model includes two ossified parts - the vertebral body and arch, as well as a pair of cartilaginous pedicles that connect them (Figure 5). Reflecting osteometric measurements, there is a notable increase in the size of the lumbar vertebrae at this developmental stage. The vertebral body remains sector-shaped with more pronounced rounded edges and features several notches. The vertebral arch has grown larger and extends downwards compared to earlier models. The arch's elongated pedicles, comprised of cartilaginous tissue and bone, frame a significantly bigger vertebral foramen in conjunction with the arch. Growth in the transverse processes is particularly dynamic in this age, a detail that was accurately incorporated into the model. The spinous processes remain faintly delineated.

Postural development is vigorous during these years, and the lumbar lordotic curve is beginning to be well visible in the 3D model of the child's lumbar spine (Figure 5E).

3D vertebral models of group 4 - Middle childhood (ages 4-7)

**Figure 6:**
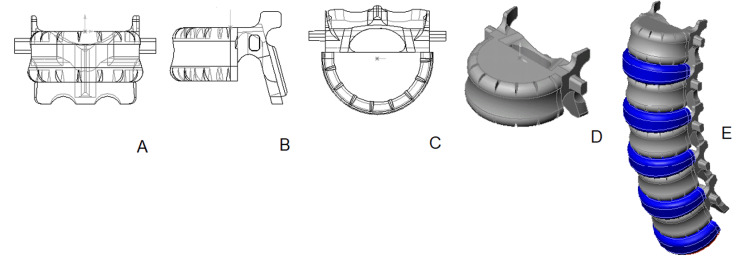
3D model of the lumbar vertebra and spine of group 4: A – anterior view; B – lateral view; C – superior view; D – isometric projection; E – assembled lumbar spine. Color scheme: gray - bone, light blue - cartilage, navy blue - intervertebral discs, red - upper endplate S1.

The 3D model representing the lumbar spine of children aged 4-7 years is a single structure, reflecting the anatomical reality that, by this age, the vertebral body and the arch have typically fused into a single vertebra. In this model, the vertebral body takes on the robust form of a cylinder sector with notably rounded upper and lower margins that exhibit several notches. The model also features a distinct narrowing or "waist" at the midsection of the vertebral body. Solid, bony pedicles from the arch assimilate with the body, while the shape of the vertebral foramen is closer to a triangle shape. The model shows lower articular processes starting to emerge from the arch (the posterior surface of the arch transitions from a prior rectangular contour, now adorned with filigree-like notches at the sides and base). For the first time, the spinal process is visible on the posterior midline of the vertebral arch. The transverse processes are located laterally as parallelepiped structures adjoined to both sides of the vertebral arch (Figure 6). In crafting a 3D model of the lumbar spine, data regarding the average dimensions of the vertebrae gathered from osteometric analysis were taken into account (Figure 6E).

3D vertebral models of group 5 - Preadolescents (ages 8-12)

**Figure 7:**
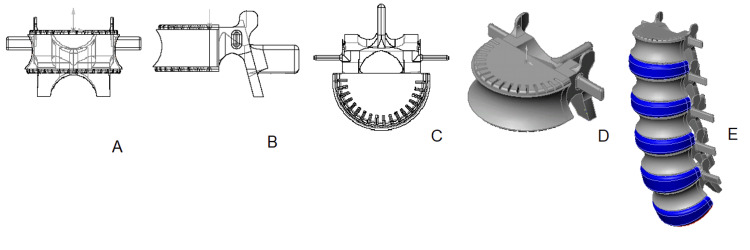
3D model of the lumbar vertebra and spine of group 5: A – anterior view; B – lateral view; C – superior view; D – isometric projection; E – assembled lumbar spine. Color scheme: gray - bone, light blue - cartilage, navy blue - intervertebral discs, red - upper endplate S1.

In developing 3D models for the lumbar vertebrae of children between 8-12 years old, osteometric data played a crucial role. The vertebral body in this model is considerably more robust and shaped like a sector of a cylinder, featuring a prominent "waist." The superior and inferior surfaces of vertebral bodies are adorned with numerous notches. A distinctive "step" cutout is modeled on the upper and lower margins, a unique characteristic of the vertebrae in this age (Figure 7), related to specific ossification patterns of vertebral apophyses.

At this stage of development, the vertebral processes are fairly developed. In the model, the spinous process is depicted as a short, strictly horizontal plate. The superior and inferior articular processes have fully separated from the arch, and the transverse processes are prominently located on the sides of the vertebral arch. In terms of proportions, the height of the intervertebral discs is relatively small compared to the height of the vertebral bodies. Recognizing the significance of posture development during these years, we indicated it while constructing the lumbar spine model for this age group (Figure 7E).

## Discussion

The structure of the human vertebrae has undergone significant transformation throughout human development. These changes reflect the evolutionary adaptations that have supported bipedal locomotion, enhanced flexibility, and increased load-bearing capacity to accommodate the demands of standing upright and the associated gravitational forces. Initially, the vertebrae would have been designed for a quadruped stance and locomotion, offering support and mobility appropriate for a horizontal backbone. As early humans adopted an upright posture, the vertebrae became more robust and complex in structure, providing the necessary support for a vertical spine.

This evolution included a shift in the shape of the lumbar vertebrae to better support the lower back, the development of a more pronounced lumbar curvature to maintain balance, and adaptations in the size and orientation of the facets joints to facilitate a wider range of motion while walking upright. In infants and young children, the vertebrae continue to undergo significant changes. They develop from cartilaginous structures to more solid bone as a part of the normal growth process. The secondary ossification centers emerge, grow, and eventually fuse with the primary centers, contributing to the final shape and size of the adult vertebrae [24]. Our research reflects these changes: created models vary the vertebral body’s size and shape based on previously done osteometric studies of the real vertebrae. Our 3D models also contain non-ossified components from cartilage tissue, which reflects different stages of vertebral development in ontogenesis.

Solid-state 3D models offer high realism and accuracy, providing a tangible representation of complex structures or objects that may be difficult to visualize in traditional 2D formats [18, 25]. Our models can be used for the creation of applications in virtual or augmented reality in medical or patient education. Users can virtually interact with solid-state 3D models, allowing for hands-on exploration and manipulation. This can enhance learning experiences and facilitate a better understanding of spatial relationships. Solid-state 3D models enable viewers to examine objects from multiple angles, helping them visualize and understand complex concepts more effectively than flat images or drawings.

Over the past decade, several studies have employed finite element modeling (FEM) to diagnose and design treatments for various abnormalities [11-14, 26], including vertebral surgery. 3D models can be used to effectively communicate ideas, designs, or concepts to other researchers and physicians. They can convey information more clearly and engagingly than traditional 2D representations. Solid-state 3D models can be customized to specific requirements, allowing for the creation of prototypes or models tailored to individual patient needs. This can be particularly useful in traumatology and orthopedics for planning surgery. They can also be converted into files for 3D printing [16, 18].

In pediatrics, some individual studies were performed to evaluate different approaches in abnormal spinal curves using 3D spinal models. Their findings indicate that solid-state 3D models and finite-element analysis can be very useful in finding the correct forces to reduce scoliosis as well as lordotic and kyphotic curvatures. These studies suggest that using a mixed approach of transverse and vertical forces could optimize the design of braces for correcting scoliosis with minimal adverse effects on the natural spinal curves [27, 28]. Our models can be modified for a more general investigation of these forces, depending on the age of the patient.

While initially requiring an investment in technology and equipment, solid-state 3D models can be cost-effective in the long run, as they can be reused, modified, and shared without the need for constant reproduction. As the technology continues to evolve, the barriers to creating and using 3D models are decreasing, making them more widely available for various purposes.

## Conclusions

In this study, pediatric solid-state vertebral models were created based on detailed osteometric data, which covers the notable gap in 3D modeling of the spine. Details of the growth patterns and morphological shapes of the lumbar vertebra at the different age groups were meticulously utilized. Created pediatric solid-state vertebral 3D models can be utilized in developing virtual or augmented reality applications and for medical research. Users can interact with models, allowing virtual exploration and manipulation, enhancing learning experiences and facilitating a better understanding of spatial relationships. These solid-state 3D models allow finite element analysis and can be used for further research to calculate internal relative deformations and stress distribution under different conditions.
